# Supplementary material for: WordCommentsAnalyzer: A windows software tool for qualitative research
Source: F1000Res. 2018 Sep 4;7:536. Originally published 2018 May 3. [Version 2] doi: 10.12688/f1000research.14819.2 (PMC6137411; doi:10.12688/f1000research.14819.2)
Supplement: Supplementary file 3 [file f1000research-7-17614-s0002.tgz › b2a79496-4ada-47c4-9682-a99ce867836d.zip!/Eight Iranian Health Journals/Salmand_Iranian Journal of Ageing _33.docx]

© Salmand_Iranian Journal of Ageing . This work is licensed under CC BY-NC 4.0 (https://creativecommons.org/licenses/by-nc/4.0/).

Volume & Issue: دوره 5 - شماره 2

Title: وضعیت و عوامل غیر‌رژیمی تغذیه در سالمندان مبتلا به نارسایی قلبی

http://salmandj.uswr.ac.ir/article-1-289-fa.html

اهداف: هدف مطالعه حاضر، ارزیابی وضعیت تغذیه و شناسایی مهمترین بخشهای پرسشنامه ارزیابی کوتاه تغذیه ای(MNA)  برای تسریع در شناخت خطرهای تغذیه ای در سالمندان مبتلا به نارسایی قلبی بود. مواد و روش‌ها: مطالعه حاضر، از نوع مقطعی توصیفی – تحلیلی است و جامعه آماری شامل تمامی سالمندان مبتلا به نارسایی قلبی بستری در مرکز آموزشی، تحقیقاتی و درمانی قلب و عروق شهید رجایی بود که 225 سالمند بیمار به صورت غیرتصادفی انتخاب شدند. از پرسشنامه ارزیابی کوتاه تغذیه ای (18 گویه ای) برای درجه بندی وضع تغذیه سالمندان استفاده شد. یافته‌ها: نتایج نشان دادند که 9/8% از سالمندان، مبتلا به سوء تغذیه و 80/9% از آنها در معرض سوء تغذیه و 9/3% خوب تغذیه شده بودند. نتیجه‌گیری: با بررسی عوامل مرتبط با وضعیت تغذیه سالمندان مبتلا به نارسایی قلبی مشخص شد که سابقه زخم بستر، توانایی سالمند در غذا خوردن، ارزیابی وضعیت سلامت خود و احساس شخصی در مورد وضعیت تغذیه خود، از عوامل موثر در وضعیت تغذیه سالمندان مبتلا به نارسایی قلبی است. با توجه به درصد بالای سالمندان در معرض یا مبتلا به سوء تغذیه در بیمارستان، ضرورت انجام مداخلات تغذیه ای به چشم می خورد.

Title: اثربخشی درمان مرور زندگی بر کاهش شدت افسردگی زنان سالمند

http://salmandj.uswr.ac.ir/article-1-286-fa.html

اهداف: هدف از انجام این پژوهش، بررسی اثربخشی درمان مرور زندگی بر کاهش شدت افسردگی زنان سالمند یزد بود. مواد و روش‌ها: پژوهش حاضر از مطالعات نیمه آزمایشی با طرح پیش آزمون–پس آزمون با گروه کنترل بود. جامعه آماری شامل سالمندان زن ساکن آسایشگاه های استان یزد بود. از بین سه آسایشگاه سالمندان موجود در استان یزد، آسایشگاه، فاطمه الزهرا تفت به صورت تصادفی انتخاب شد. پس از اجرای فرم 15 سوالی آزمون GDS بر 30 نفر از سالمندان زن ساکن آسایشگاه 16 نفر از کسانی که نمره 8 یا بالاتر از این آزمون گرفته بودند، به طور تصادفی در دو گروه آزمایش (8 نفر) و گروه کنترل (8 نفر) جایگزین شدند. برای گروه آزمایش 6 جلسه گروه درمانی مرور زندگی در طول 6 هفته بر اساس طرح درمانی پژوهش اجرا شد. یک هفته پس از اتمام جلسات درمانی، پس آزمون افسردگی GDS-15 برای هر دو گروه اجرا شد و نتایج با آزمون تحلیل کوواریانس مورد بررسی قرار گرفت. یافته‌ها: تحلیل کوواریانس نتایج پیش آزمون و پس آزمون افسردگی، (GDS-15) بیانگر تفاوت معنی دار در اندازه شدت افسردگی گروه ها بود. نتیجه‌گیری: روش درمان مرور زندگی بر کاهش شدت افسردگی زنان سالمند موثر است. این درمان در مقایسه با درمانهای سنتی، نتیجه بهتری می دهد و انجام این درمان در مراکز سالمندی توصیه می شود.

Title: مقایسه سرعت راه رفتن سالمندان با یا بدون سابقه زمین خوردن در انجام تکالیف دوگانه

http://salmandj.uswr.ac.ir/article-1-285-fa.html

اهداف: پژوهش حاضر با هدف بررسی تفاوت سرعت راه رفتن در سالمندان با / بدون سابقه زمین خوردن در شرایط انجام تکالیف دوگانه شناختی و حرکتی انجام شد. مواد و روش‌ها: تحقیق حاضر از نوع علّی‌مقایسه ای بود. بدین منظور تعداد 80 سالمند مرد از سه پارک در شهر تهران، بعد از تکمیل پرسشنامه ارزیابی هشیاری، به صورت در دسترس انتخاب شدند. آزمودنی ها شامل 40 سالمند بدون سابقه زمین خوردن (72/6±5 سال) و 40 سالمند با سابقه زمین خوردن (74/50±6 سال) بودند. همه آزمودنی ها آزمون سرعت راه رفتن را تحت سه شرایط تکالیف مجرد (خود آزمون)، تکالیف دوگانه شناختی–راه رفتن (اجرای آزمون همراه با شمارش معکوس اعداد تصادفی) و تکالیف دو گانه حرکتی–راه رفتن (اجرای آزمون همراه با حمل فنجان پر آب) انجام دادند. تجزیه و تحلیل با استفاده از تحلیل واریانس چند متغیری در سطح P<0/05 انجام شد. یافته‌ها: نتایج نشان داد که تفاوت معناداری بین گروه های سالمند با و بدون سابقه زمین خوردن در متغیر خطر زمین خوردن وجود داشت (P=0/0005) به طوری که سالمندان بدون سابقه زمین خوردن در همه آزمونها وضعیت بهتری داشتند. نتیجه‌گیری: به طور کلی می توان آزمون سرعت راه رفتن تحت شرایط تکلیف دو گانه شناختی و حرکتی را به عنوان آزمونی برای تمایز سالمندان با و بدون سابقه زمین خوردن برای فاکتور مهم الگوی راه رفتن به کار برد.

Title: ارتباط ویژگی‌های شخصیتی با شدت علائم افسردگی در سالمندان زن فعال و غیرفعال شهر تهران

http://salmandj.uswr.ac.ir/article-1-287-fa.html

اهداف: هدف از پژوهش حاضر بررسی ارتباط ویژگی های شخصیتی با افسردگی در سالمندان زن فعال و غیر فعال شهر تهران بود. مواد و روش‌ها: مطالعه حاضر توصیفی – همبستگی است. بدین منظور، 188 سالمند زن (94 سالمند فعال و 94 سالمند غیر فعال) شهر تهران با دامنه سنی 63-82 سال به روش نمونه گیری در دسترس، به عنوان نمونه آماری انتخاب شدند. ابزار پژوهش شامل پرسشنامه خصوصیات فردی، پرسشنامه افسردگی بک و پرسشنامه ویژگی های شخصیتی نئو بود. برای تجزیه و تحلیل داده ها از آمار توصیفی نظیر میانگین و انحراف معیار و از آمار استنباطی نظیر آزمون t مستقل، همبستگی پیرسون و تحلیل رگرسیون چند متغیره به روش ورود استفاده شد. یافته‌ها: نتایج پژوهش نشان داد که بین نمرات ویژگی های شخصیتی و افسردگی سالمندان زن فعال با سالمندان زن غیر فعال تفاوت معنی داری وجود دارد (P<0/05). همچنین بین نمره افسردگی با نمرات نوروتیسم، برون گرایی، مقبولیت اجتماعی و وظیفه شناسی در سالمندان زن فعال و بین نمره افسردگی با نمرات مقبولیت اجتماعی و وظیفه شناسی در سالمندان زن غیر فعال همبستگی معنی داری وجود دارد (P<0/05). نتایج تحلیل رگرسیون چند متغیره نیز نشان داد که متغیر مقبولیت اجتماعی و برون گرایی در زنان سالمند فعال و متغیر وظیفه شناسی در زنان سالمند غیر فعال دارای رابطه خطی با متغیر معیار (افسردگی) بوده و می تواند تغییرات مربوط به آن را پیش بینی نماید. نتیجه‌گیری: بر اساس نتایج تحقیق حاضر، فعالیت بدنی و ورزش می تواند به تقویت ویژگی های شخصیتی مثبت نظیر برون گرایی، مقبولیت اجتماعی و کاهش افسردگی در سالمندان کمک کند.

Title: ارتباط بین سندرم متابولیک و لپتین سرم در سالمندان

http://salmandj.uswr.ac.ir/article-1-283-fa.html

اهداف: بررسی ارتباط بین سندرم متابولیک، مقاومت به انسولین و لپتین در زنان سالمند هدف پژوهش حاضر است. مواد و روش‌ها: در این مطالعه مقطعی، از میان 225 فرد 60 ساله یا بالاتر ساکن آسایشگاه کهریزک که به صورت تصادفی جزو لیست احتمالی شرکت کنندگان در مطالعه قرار گرفتند، 133 نفر (56 مرد و 77 زن) به روش نمونه گیری تصادفی خوشه ای وارد مطالعه شدند. فشارخون سیستولی، دیاستولی و مقادیر ناشتای تری گلیسرید، کلسترول تام، HDL و لپتین سرم اندازه گیری شد. یافته‌ها: میانگین سنی مردان و زنان سالم به ترتیب 76/88±6/56 و 79/49±7/96 سال و در مبتلایان به سندرم متابولیک 71/00±5/19 و 77/94±7/24 سال بود. از بین 133 فرد مورد مطالعه 15 مرد و 34 زن به سندرم متابولیک مبتلا بودند. در مردان و زنان، همبستگی معنادار قوی بین لپتین سرم و سندرم متابولیک و نمایه توده بدن وجود داشت (P<0/001). از میان اجزای سندرم متابولیک تنها دور کمر با لپتین سرم همبستگی مشابهی در هر دو جنس نشان داد (P<0/001، در مردان r=0/68 و در زنان r=0/48). در مردان و زنان ارتباط بین سندرم متابولیک و لپتین سرم پس از مشابه سازی اجزای سندرم متابولیک همبستگی معنادار نشان داد (P<0/05). اما پس از مشابه سازی بر اساس دور کمر این ارتباط معنادار نبود. نتیجه‌گیری: مطالعه حاضر نشان داد که ارتباط بین سندرم متابولیک و لپتین احتمالا به واسطه دور کمر است.

Title: تأثیر موسیقی‌درمانی بر میزان افسردگی و احساس تنهایی سالمندان

http://salmandj.uswr.ac.ir/article-1-288-fa.html

اهداف: پژوهش حاضر با هدف بررسی اثر موسیقی درمانی بر کاهش افسردگی و احساس تنهایی سالمندان انجام شد. مواد و روش‌ها: پژوهش حاضر مطالعه ای شبه تجربی با روش پیش آزمون و پس آزمون با گروه کنترل بود. 38 سالمند (20 مرد و 18 زن) با روش نمونه گیری در دسترس از شهرستان سیرجان انتخاب شدند و به تصادف در دو گروه آزمایش و کنترل قرار گرفتند. ابزارهای مورد استفاده پرسشنامه افسردگی سالمندان، پرسشنامه احساس تنهایی و بسته آموزشی موسیقی درمانی بود. مداخله شامل 13 جلسه موسیقی درمانی بود. یافته‌ها: نتایج پژوهش حاکی از آن است که افسردگی سالمندان پس از مداخله کاهش یافته است. همچنین احساس تنهایی زنان در قسمت ارتباط با دوستان و نشانه های عاطفی تنهایی، بهبود معنادار داشته است اما بر احساس تنهایی مردان تاثیر معنادار نداشت. نتیجه‌گیری: یافته های تحقیق نشان دادند که موسیقی درمانی می توان به عنوان راهکاری برای کاهش شدت افسردگی سالمندان به کار رود؛ تاثیر این گونه مداخلات بین افراد دو جنس می تواند متفاوت باشد؛ و این تفاوت در زمینه کاهش احساس تنهایی نیز به چشم می خورد.

Title: اثر بخشی درمان با محدودسازی بر کیفیت خواب سالمندان

http://salmandj.uswr.ac.ir/article-1-282-fa.html

اهداف: هدف از پژوهش حاضر، تعیین اثر درمان با محدود سازی بر کیفیت خواب سالمندان بود. مواد و روش‌ها: پژوهش حاضر، مطالعه ای نیمه تجربی بود، جامعه پژوهش شامل 5000 سالمند عضو کانون جهاندیدگان شیراز و نمونه متشکل از 50 نفر با احتساب معیارهای ورود و خروج بودند. به دنبال ریزش 25 درصد، 38 سالمند (24 زن و 14 مرد) با میانگین سنی 65 سال که مبتلا به بی خوابی اولیه و کیفیت خواب نامناسب بودند، با شاخص کیفیت خواب پیتزبورگ مورد ارزیابی قرار گرفتند. شرکت کنندگان به روش بلوک ه ای تصادفی در دو گروه مداخله (19 نفر) و کنترل (19 نفر) قرار گرفتند. مداخله به مدت 4 هفته، و با بسته درمانی محدودیت خواب، انجام شد. برای تجزیه و تحلیل داده ها از SPSS 16 و آزمون های آماری کلموگروف اسمیرنوف، تی زوجی و تی مستقل استفاده شد. یافته‌ها: میانگین نمره کیفیت خواب قبل از مداخله، در گروه های آزمایش و کنترل به ترتیب 14/21 و 13 بود که پس از مداخله به مقادیر 11/26 و 12/78 تغییر کرد. تفاوت میانگین نمرات کیفیت خواب پس از مداخله از لحاظ آماری معنادار (P=0/038) بود. نتیجه‌گیری: مطالعه حاضر نشان داد درمان با محدودیت خواب می تواند منجر به ارتقا کیفیت خواب در سالمندان شود. لذا آموزش این مداخلات در قالب آموزش ضمن خدمت به کادر درمان و به خصوص پرستاران شاغل در حیطه سالمندی توصیه می شود.

Title: فراوانی آنمی در سالمندان مبتلا به نارسایی سیستولیک قلبی

http://salmandj.uswr.ac.ir/article-1-284-fa.html

اهداف: هدف از مطالعه حاضر، تعیین فراوانی آنمی در سالمندان مبتلا به نارسایی سیستولیک قلبی بود. مواد و روش‌ها: مطالعه حاضر، از نوع توصیفی گذشته نگر بود که در آن اطلاعات دموگرافیک، آزمایشگاهی و اکوکاردیوگرافی 154 پرونده پزشکی متعلق به سالمندان مبتلا به نارسایی سیستولیک قلبی (1382 تا 1387) مورد بررسی قرار گرفت. در این مطالعه هموگلوبین کمتر از 13 گرم در دسی لیتر در مردان و کمتر از 12 گرم در دسی لیتر در زنان آنمی تعریف شد. از توزیع فراوانی، میانگین و انحراف معیار و آزمون تی برای گروه های مستقل و ضریب همبستگی پیرسون برای تجزیه و تحلیل داده ها استفاده شد. یافته‌ها: نتایج نشان داد میزان شیوع آنمی در سالمندان مبتلا به نارسایی سیستولیک قلبی (42/9%) بود. تفاوت آماری معنی داری بین سطح هموگلوبین خون در بیماران مبتلا به نارسایی سیستولیک قلبی دارای نارسایی مزن راه های هوایی (P=0/001) وجود داشت و سطح هموگلوبین خون با سن (P=0/014) و کسر جهشی بطن چپ (P=0/022) ارتباط معنی داری داشت. نتیجه‌گیری: آنمی در سالمندان مبتلا به نارسایی سیستولیک قلبی شایع بوده و آنمی ممکن است با سن، کسر جهشی بطن چپ و بیماریهای مزمن انسدادی ریه ارتباط داشته باشد. تحقیق بیشتر در جهت شناسایی مکانیسم های آنمی مورد نیاز است.
